# Piezoelectric-assisted removal of a benign fibrous histiocytoma of the mandible: An innovative technique for prevention of dentoalveolar nerve injury

**DOI:** 10.1186/1746-160X-7-20

**Published:** 2011-10-31

**Authors:** Maximilian EH Wagner, Majeed Rana, Wolfgang Traenkenschuh, Horst Kokemueller, André M Eckardt, Nils-Claudius Gellrich

**Affiliations:** 1 Department of Cranio-Maxillo-Facial Surgery, Hannover Medical School, Germany; 2 Department of Pathology, Hannover Medical School, Germany

**Keywords:** Piezosurgery, benign fibrous histiocytoma, mandibular tumor, dentoalveolar nerve, atraumatic bone surgery

## Abstract

In this article, we present our experience with a piezoelectric-assisted surgical device by resection of a benign fibrous histiocytoma of the mandible.

A 41 year-old male was admitted to our hospital because of slowly progressive right buccal swelling. After further radiographic diagnosis surgical removal of the yellowish-white mass was performed. Histologic analysis showed proliferating histiocytic cells with foamy, granular cytoplasm and no signs of malignancy. The tumor was positive for CD68 and vimentin in immunohistochemical staining. Therefore the tumor was diagnosed as primary benign fibrous histiocytoma. This work provides a new treatment device for benign mandibular tumour disease. By using a novel piezoelectric-assisted cutting device, protection of the dentoalveolar nerve could be achieved.

## Background

According to the WHO histological classification of tumors, primary benign fibrous histiocytoma (BFH) of bone is defined as a benign lesion composed of spindle-shaped fibroblasts, arranged in a storiform pattern, with a variable admixture of small, multinucleated osteoclast-like giant cells. Foamy cells (xanthoma), chronic inflammatory cells, stromal haemorrhages and haemosiderin pigment are also commonly present [1]. According to this classification, there are less than 100 reported cases of BFH worldwide and only six reported cases in the mandible [2-7]. It is usually found in long bones, especially femur and tibia, and the pelvic bone, but may occur in virtually any bone. However, the precise removal especially in close vicinity to nerval structures is challenging. In our case resection of a mandibular tumor by preventing injury to the dentoalveolar nerve is difficult.

The presented case enlarges the indications for the use of ultrasonic devices in tumor surgery and thus emphasizes the beneficial effects of this technique in bone cutting close to nerval structures.

## Materials and methods

**Figure 1:**
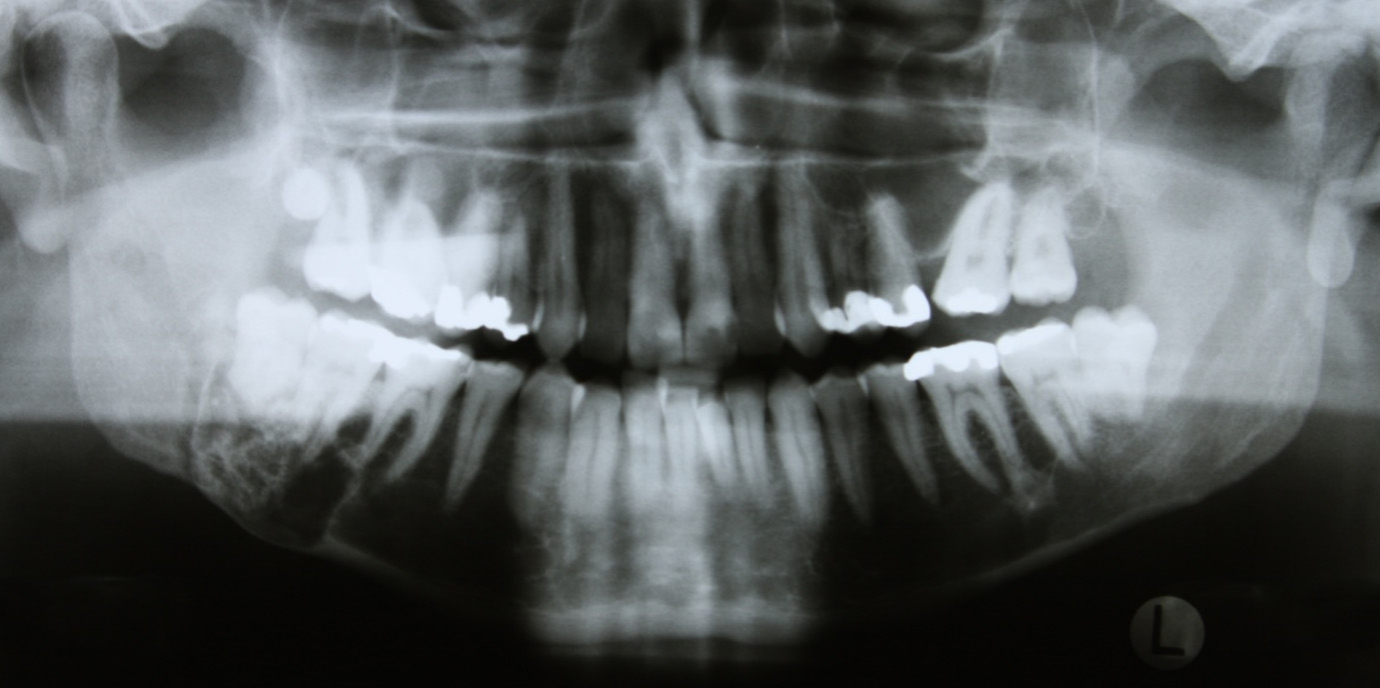
**Preoperative panoramic radiograph**.

A 41-year old Caucasian man was referred to our clinic for evaluation of a slowly progressive swelling of his right mandible. A panoramic radiograph (Figure 1) showed a well-demarcated multilocular radiolucent lesion with a reactive hyperostotic border in the right mandibular molar region. No other symptoms had been noted before.

**Figure 2:**
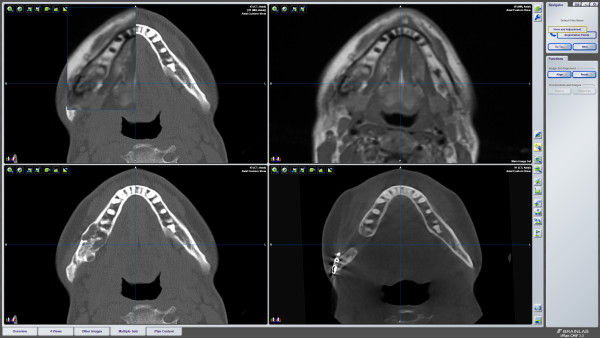
**Preoperative CT and MRI scans showing the heterogeneous lesion in the right mandible with no vascular signs**.

A computed tomography (CT) scan was obtained, which showed a heterogeneous soft-tissue mass (Figure 2). There was vertical expansion more prominent of the lingual side with thinning of the cortex and two small spots of cortical destruction. No lymph node involvement was observed. A magnetic resonance imaging (MRI) scan was performed to exclude the presence of a haemangioma prior to osseous biopsy (Figure 2).

**Figure 3:**
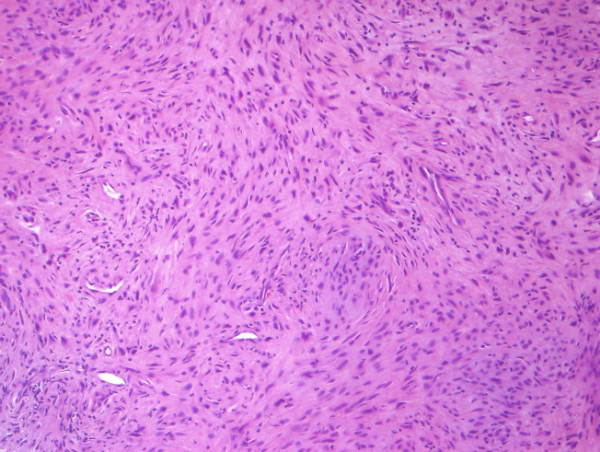
**Histopathological examination of the obtained tissue showing spindle-shaped fibroblasts, arranged in a storiform pattern (hematoxylin-eosin-staining, magnification 25×)**.

**Figure 4:**
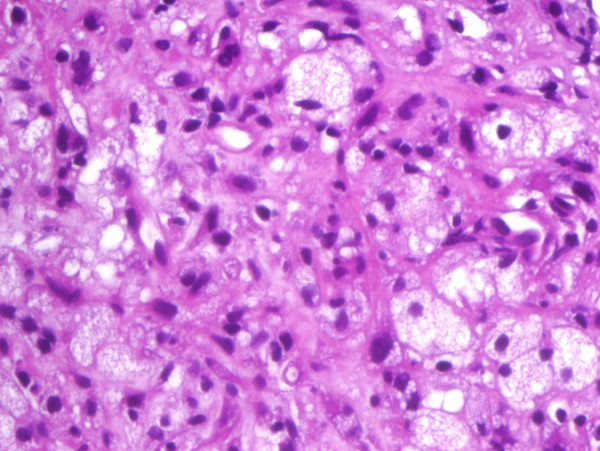
**While in other parts of the specimen proliferating histiocytic cells with foamy, granular cytoplasm and no signs of malignancy dominate (hematoxylin-eosin-staining, magnification 100×)**.

**Figure 5:**
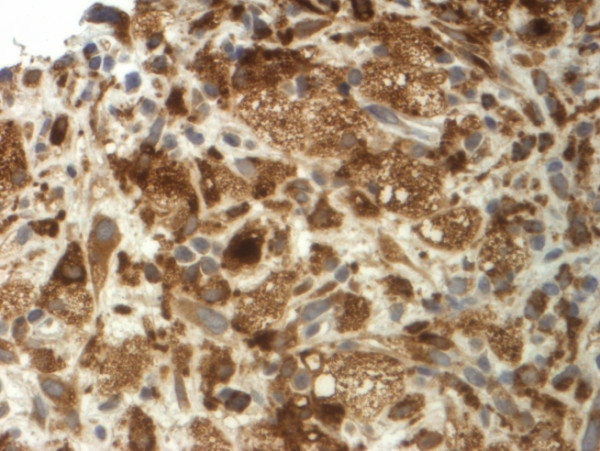
**Immunohistochemical staining positive for CD68 (magnification 100×)**.

**Figure 6:**
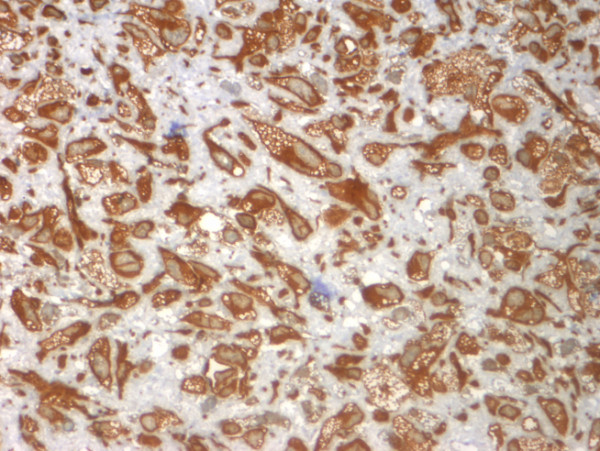
**mmunohistochemival staininga also positive for vimentin (magnification 100×), and negative for sm-actin, desmin, cytokeratin, S-100 protein or CD-56**.

Histopathological examination (Figure 3, 4) and immunohistochemical staining (Figure 5, 6) confirmed the diagnosis of primary benign fibrous histiocytoma.

**Figure 7:**
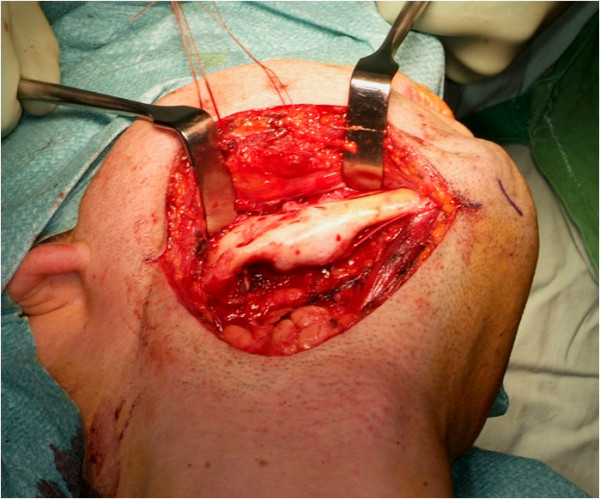
**Intraoperative image of the original mandible**.

**Figure 8:**
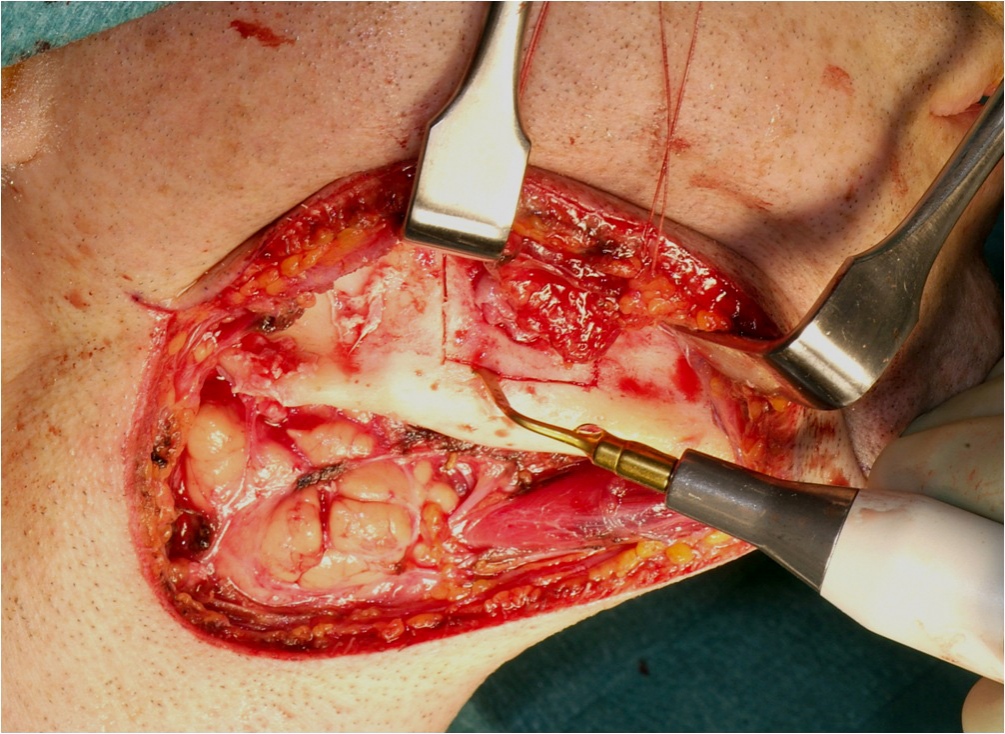
**Intraoperative image showing the removal of the bone with the piezosurgery device**.

**Figure 9:**
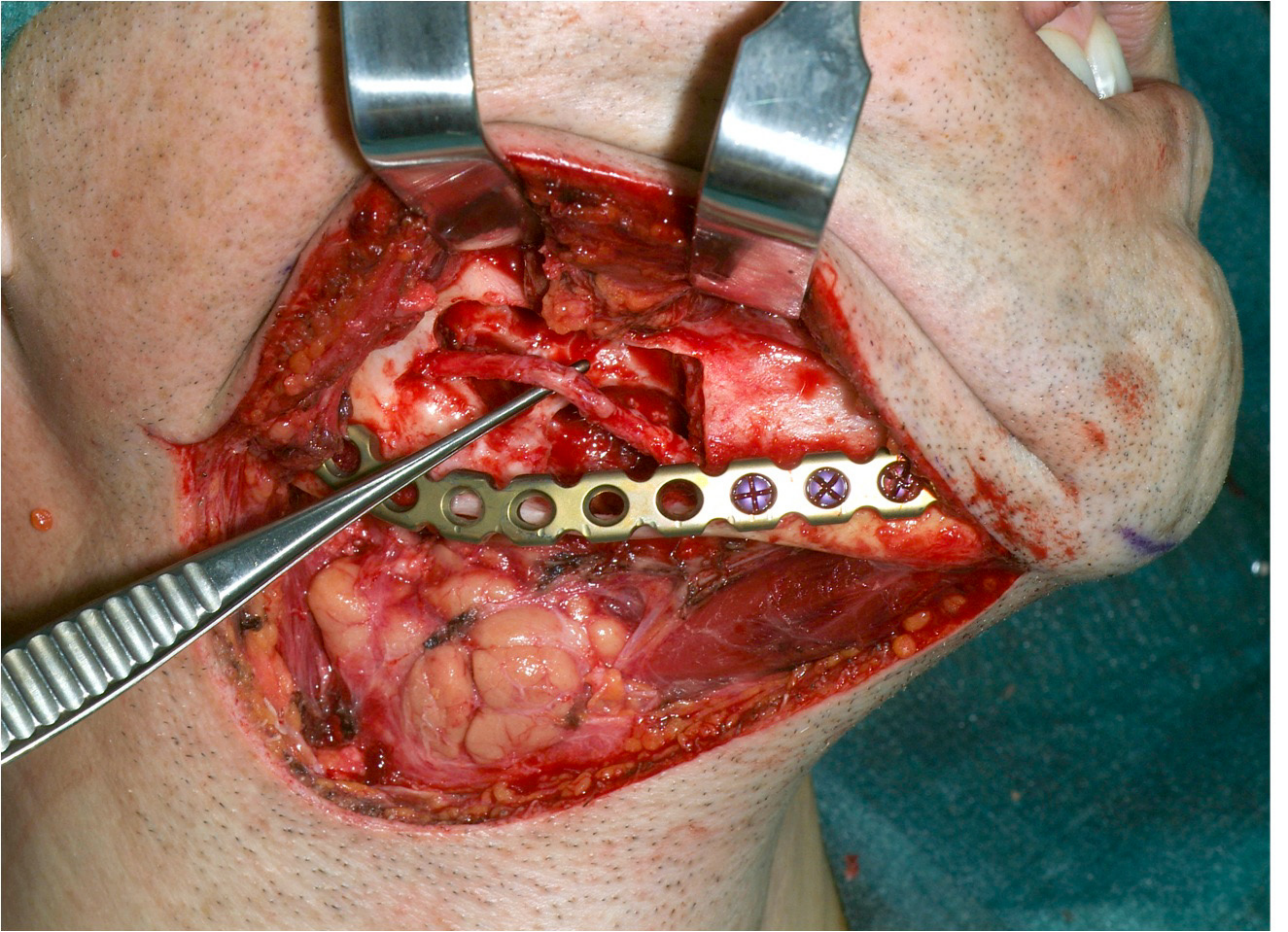
**Intraoperative image after removal of the tumor**.

The patient was treated definitely via an extraoral submandibular approach (Figure 7). Simple cyst-like excochleation of the tumor in one piece was not possible due to different consistencies of the lesion. Rubber-like soft tissue parts of the tumor could be removed by curettage and excision, while bone-like hard tissue parts had to be removed using a bone drill. To prevent any nerve damage, bone-like hard tissue parts in the vicinity of the dentoalveolar nerve were removed exclusively by using the piezoelectric device (Figure 8). Despite the cortical destruction of lingual and buccal bone, the surrounding tissue was not affected. The lower rim of the right mandible could be preserved, stabilized with a osteosynthesis plate for fracture prevention. In order to achieve complete resection of the tumor, the teeth 46 and 47 were extracted and neurolysis of the inferior alveolar nerve was performed (Figure 9).

The neurological analysis was performed bilaterally. It was used to evaluate nerve dysfunctions. The skin of the mental region, upper and lower lip were checked using a cotton test for touch sensation, a pinprick test using a needle for sharp pain and a blunt instrument for testing pressure. Additionally, a two point discrimination test was executed on these regions. The same procedure was accomplished for the lower lip and the mental nerve skin region. The results were recorded on a score that ranges between 0 and 13, with 13 being the worst neurological score. The neurological score was assessed at 4 points in time: on the 1^st ^(T1 = 9), the 10^th ^(T2 = 7), the 22^nd ^(T3 = 3), the 184^th ^(T4 = 1) postoperative day.

Piezosurgery^® ^(Mectron^®^-Germany, Cologne, Germany) is an ultrasound device introduced in medical practice in 1988 for different procedures in application to hard tissues, including periodontal surgery, periapical surgery,[8,9] the removal of impacted teeth, implant surgery for facilitating bone ridge expansion or in bone regeneration techniques,[10,11] inferior dental nerve lateralization and transpositioning. Furthermore ultrasound has lately been used for osteotomies as well as for dental implant bone preparation and thus presents an additional option for cutting bone beside the classic osteotomy techniques using rotating burs or oscillating saws [12]. With this new option, the bone is cut almost without pressure through piezoelectrically induced oscillations. Micro-movements of 60-200 μm ensure that only the mineralized hard tissue is cut. The frequency of the oscillations applied in osteotomies lies between 22 and 29 kHz. This makes it possible to reliably prevent damage to soft tissue and nerve tissue during an osteotomy [10,13]. Trauma to these types of tissue is only likely to occur at frequencies of 50 kHz or more [14,15].

## Discussion

A primary benign fibrous histiocytoma in the mandible is extremely rare with only six reported cases in the literature [2-7].

The etiology of BFH is not yet clear. It may be a neoplasm consisting of fibroblasts and histiocytic-like cells [16] or a regression phenomenon of giant cells tumors [6]. BFH is mainly found in the pelvic bone, femur and tibia. Patients often report a history of pain or swelling over a long period of time, sometimes years. A sclerotic rim around the osteolytic defect is common [17].

The histologic appearance of BFH is identical to non-ossifying fibroma, making a clinical radiographic evaluation indispensable. The non-ossifying fibroma typically occurs during growth. BFH on the other hand is found in older patients, presenting with swelling or pain but usually no presence of complicating fractures. Non-ossifying fibroma is limited to the metaphysis of mainly the lower extremities, whereas BFH is found in the epi- or diaphysis or in flat bones [17].

To distinguish BFH from giant cell tumors can be challenging. On the one hand giant cells can be numerous in BFH, even if the mononuclear cell component is more spindled and associated with collagen formation [18]. On the other hand focal or extended fibrous tissue with lipid-bearing histiocytes can be found in giant cell tumor specimens [19,20]. It was suggested to differentiate the two diseases radiologically due to the fact that most giant cell tumors are very much vascularized. The presence of a sclerotic rim in BFH could also be used to differentiate these two diseases [21].

One of the microscopic features is the presence of lipid-bearing histiocytes - also called xanthoma cells - sometimes dominating the histological picture in BFH.

As there are at least three reports of xanthomatous lesions in the mandible [22-24], a comparison with BFH seems reasonable. Xanthomas of the bone are tumor-like accumulations of lipid-bearing histiocytes, either in combination with hyperlipoproteinemia or as part of other lesions like BFH. Xanthomas are no tumorous proliferation of any cellular element of the bone [17]. Therefore it is not listed in the WHO histological classification of bone tumors [1]. Radiographically, xanthomas lack a sclerotic rim. In contrast to BFH, extension into the adjacent soft tissue is reported in xanthomas [25]. In our case no extension in the surrounding soft tissue was detected, although there was cortical disruption at the lingual and buccal bulging.

The prognosis for BFH seems to be excellent with almost no recurrence after complete surgical resection. Due to the dominance of the bone mass close to the dentoalveolar nerve, the piezoelectric unit was a usefull tool to prevent nerve injury.

Ultrasonic waves are used in oral and maxillofacial surgery for various diagnostic and therapeutic procedures. They are applied in diagnostics, endodontics, the removal of calculus from the teeth and, most recently, osteotomies [26-29]. Depending on the indication, the oscillation amplitude and frequency vary in accordance with the power transmitted to the tissue. Special presets are indicated for bone cutting procedures.

In the presented case the neurological scores from T1 to T4 demonstrate no dental nerve injury. No damage to the nerve was detectable even though direct contact of the working tip with the alveolar nerve was to be assumed. This is in line with experimental in vitro studies where no damage even in direct contact to the nerve was analyzed [10].

**Figure 10:**
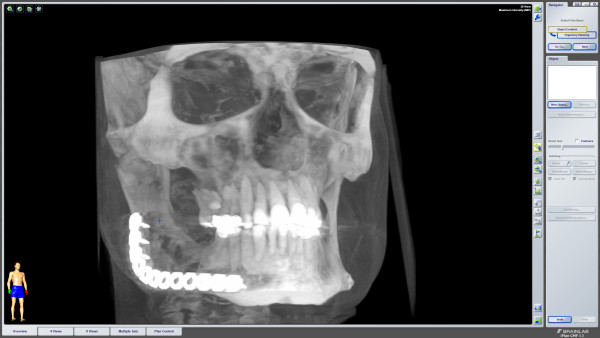
**Postoperative CBCT showing the defect and the titanium plate**.

Follow-up examinations were obtained 3 and 6 months after surgery with no clinical and radiological evidence of recurrence (Figure 10).

## Conclusions

The purpose of the present article was to show the advantages of the piezoelectric-assisted surgical removal of a rare benign fibrous histiocytoma of the mandible and give a precise description of the experience with protecting dentoalveolar nerve.

BFH must be distinguished from non-ossifying fibroma or giant cell tumors by clinical appearance as well as histopathological appearance. As far as we know, the prognosis of BFH seems to be excellent after complete removal.

There is a therapeutical potential and benefit of the Piezoelectric-assisted surgical saw in dentoalveolar surgery. Piezosurgery^® ^vibrates with a modulated ultrasonic frequency. Because the vibration frequency of Piezosurgery is optimal for mineralized tissue it does not cut soft tissue and therefore provides a technique for osteotomy to remove bony mass of the mandible and prevent anatomic soft tissue injuries like dentoalveolar nerve even in rare and complicated cases like this.

## Competing interests

The authors declare that they have no competing interests.

## Authors' contributions

MW and MR contributed equally to this work. MW, MR, WT, HK, AME and NCG conceived of the study and participated in its design and coordination. MW and MR drafted the manuscript. AME and NCG were involved in revising the manuscript. All authors read and approved the final manuscript.

## Consent statement

Written informed consent was obtained from the patient for publication of this case report and accompanying images. A copy of the written consent is available for review by the Editor-in-Chief of this journal.

## Funding

The article processing charges are funded by the Deutsche Forschungsgemeinschaft (DFG), "Open Access Publizieren".
